# HCV Integrated Care: A Randomized Trial to Increase Treatment Initiation and SVR with Direct Acting Antivirals

**DOI:** 10.1155/2017/5834182

**Published:** 2017-07-27

**Authors:** Erik J. Groessl, Lin Liu, Marisa Sklar, Samuel B. Ho

**Affiliations:** ^1^VA San Diego Healthcare System, San Diego, CA, USA; ^2^University of California, San Diego, San Diego, CA, USA

## Abstract

**Background and Aims:**

Psychiatric or substance use disorders are barriers to successful HCV antiviral treatment. In a randomized, controlled trial (RCT), the effects of HCV Integrated Care (IC) for increasing treatment rates and sustained viral response (SVR) were studied with direct acting antivirals (DAA).

**Methods:**

In 2012-13, VA patients, whose screening was positive for depression, PTSD, or substance use (*N* = 79), were randomized to IC or Usual Care (UC). IC consisted of brief psychological interventions and case management. The primary endpoint was SVR among patients followed for an average of 16.6 months.

**Results:**

42% of the study participants were previously homeless and 79% had HCV genotype 1. Twice as many IC participants (45%) initiated treatment compared with UC participants (23%) (*χ*^2^ = 4.59, *p* = 0.032). Among those treated, SVR rates did not significantly differ (IC: 12/18 = 67%; UC: 5/9 = 55%; *p* = 0.23). Among all randomized participants, IC participants trended toward better SVR rates (30.0% versus 12.8% in UC; *p* = 0.07).

**Conclusions:**

Although first-generation DAAs are no longer used, this smaller RCT helps confirm the results of a larger multisite RCT showing that Integrated Care results in higher treatment initiation and SVR rates among HCV-infected persons with comorbid psychological disorders. Integrated mental health services can facilitate treatment among the most challenging HCV patients, many of whom have not been successfully treated. This trial is registered with ClinicalTrials.gov number NCT00722423.

## 1. Introduction

Almost three million people remain chronically infected with hepatitis C virus (HCV) in the US [1, 2] with 80 million infected globally [3]. The medical consequences of HCV are still increasing because of the 20–40-year lag time between initial infection and health complications [4]. Thus, it is a crucial time for both identifying undetected HCV cases and treating large numbers of HCV-infected people with antiviral treatments [5–7].

High SVR rates (>90%) in clinical trials [8] and reduced side effect profiles of the latest direct acting antiviral (DAA) regimens have led some to declare this as a “golden era” in the fight to eradicate HCV [5]. In the past, however, SVR rates found in HCV antiviral treatment clinical trials are often diminished outside of controlled trial settings [9] and among patients who face more barriers to successful treatment [10]. Evidence suggests that psychiatric comorbidity and/or substance use disorders (SUD) continue to be barriers to treating more people. A recent VA medical center study found that 45% of current HCV patients were deemed poor candidates for interferon-free treatment based on their comorbidity profiles (psychiatric and substance use disorders) [11]. Similarly, another medical center reported that 30% of treatment-eligible, high priority HCV patients most at risk for advanced fibrosis were unable or unwilling to engage in HCV care despite outreach efforts. In that study, homelessness, substance use disorder, and other comorbidities were associated with nonengagement in care [12]. Finally, some HCV-infected persons with active or recent SUDs have been considered ineligible for Medicaid payments for interferon-free medications [13, 14].

To address ongoing barriers to treatment success with more efficacious DAA treatments [15], researchers have developed various interventions designed to improve care access, educate patients, improve treatment adherence, increase treatment initiation and completion rates, and ultimately improve treatment success. One study used patient navigators and multidisciplinary teams to assess risk, educate and prepare patients for treatment, and coordinate medications to improve adherence among HCV-infected patients with greater needs [16]. Treatment initiation increased 2- to 3-fold and on-site patients receiving more services had higher odds of initiating treatment [16]. Another recent study used a multidisciplinary team focused on educating patients before antiviral treatment and facilitating medication adherence in an HMO setting [17]. The study lacked a comparison group but obtained SVR rates in an outpatient HMO setting similar to those found in highly controlled clinical trials.

These recent studies build upon previous research on HCV self-management [18–21] and the Hepatitis C Integrated Care Program [22–25]. Integrated Care (IC) emphasizes patient-provider collaboration and involves the delivery of case management, education, and brief psychological interventions within the HCV clinic, promoting access and improved care. Our prior multisite RCT demonstrated that the HCV Integrated Care Program resulted in significant increases in treatment initiation and SVR rates with pegylated interferon plus ribavirin regimens [24]. The objective of the current study was to determine if the prior multisite trial results could be replicated with* first-generation* DAA triple therapy regimens (boceprevir/telaprevir, pegylated interferon, and ribavirin) in a small RCT. We hypothesized that HCV patients receiving Integrated Care would have higher treatment initiation and SVR rates than patients receiving care as usual.

## 2. Methods

The study was approved by the University of California, San Diego institutional review board. All project activities comply with Ethical Principles for Medical Research Involving Human Subjects outlined in the Helsinki Declaration.

### 2.1. Study Design

**Figure 1:**
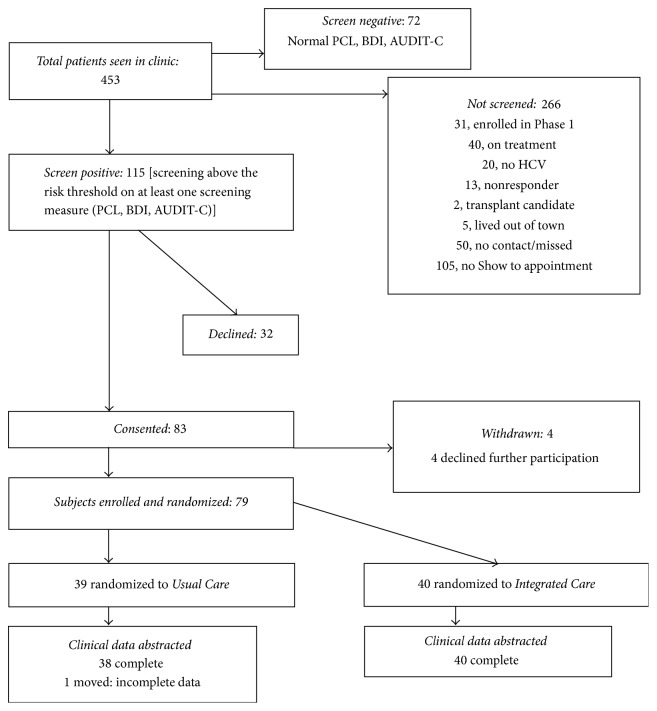
Patient enrollment and randomization.

The study was conducted at a single VA medical center with an established HCV clinic staffed by experienced physicians. Patients attending this HCV clinic were screened and recruited from January 2012 through February 2013 (Figure 1). Eligible patients were consented and randomized with a 1 : 1 ratio using online random assignment tools. The blocked randomization sequence with 20 subjects per block was concealed from the project staff that enrolled and randomized participants. A detailed description of the intervention has been published with the prior study methods [22]. A sample of 88 subjects was initially targeted, providing power of 80% to detect a difference between the groups on overall SVR rates. It was assumed that 35% and 60% of participants would be treated in the Usual Care and Integrated Care groups, respectively, and that 60% of treated patients in each group would achieve an SVR, producing 16 and 9 SVRs.

### 2.2. Study Participants

Participants were VA patients with confirmed active HCV infection (HCV PCR positive) whose screening was positive for substance use or psychiatric risk factors that were viewed as relative contraindications to antiviral treatment [26]. As part of clinical care, all patients attending the HCV clinic are asked to complete a standardized screening form. The self-administered form screened for symptoms of depression (scores ≥ 10) on the Beck Depression Inventory (BDI), posttraumatic stress disorder (endorsement ≥ 3 items) on VA Primary Care PTSD Screen (PTSD), alcohol use (AUDIT-C) (scores ≥ 4), and self-reported active drug use in prior 6 months on the Drug Use Questionnaire (excluding marijuana). In addition, clinic staff checked medical records for positive urine toxicology in 6 months prior to baseline (excluding marijuana). Criteria for exclusion included participation in the previous multisite trial of HCV Integrated Care, decompensated cirrhosis, and other significant life-threatening diseases (serious or incapacitating cancer or cardiac, renal, pulmonary, or autoimmune medical disease). Patients with prior treatment experience and patients who were nonresponders or had significant adverse events were also excluded. More details on criteria have been published previously [22].

### 2.3. Interventions

#### 2.3.1. Usual Care (UC)

Usual Care (UC) was consistent with current VA treatment guidelines for required “standard of care” [27]. The HCV clinic had a gastroenterologist and a hepatologist physician coordinating care with clinical nursing, internal medicine physicians, and a clinic psychiatrist.

The team weighed the severity of risk factors (screening measures, compliance history, and medical history) for each patient and either (a) referred them to mental health and/or substance use clinics for further assessment and treatment or (b) managed risk factors within the HCV clinic (primarily medication management) and prepared them for antiviral treatment.

#### 2.3.2. Integrated Care (IC)

Integrated Care (IC) followed a manualized protocol delivered by a mental health provider (MHP) who had a Master's degree in Marriage and Family Therapy. The MHP provided ongoing brief psychological interventions (cognitive behavioral and motivational interviewing) designed to address the specific risk factors identified at screening. Through regular individual appointments, the MHP facilitated a complete treatment evaluation, encouraged the initiation of antiviral treatment, and served as a regular contact and case manager both before and during antiviral treatment. The MHP met regularly with clinic physicians, nurses, and other mental health providers to discuss progress toward treatment. Details of the IC intervention and protocol have been published [22, 24].

### 2.4. Antiviral Treatment

Participants in both intervention arms were offered antiviral treatment following VA hepatitis C treatment guidelines [27]. The guidelines indicated that treatment candidates should demonstrate compliance with healthcare recommendations, stable psychiatric disease, and abstinence from substance use for a time period established by the clinic. All participants that initiated antiviral treatment were monitored using standard protocols for each medication regimen. The standard HCV genotype 1 antiviral medication regimens were boceprevir or telaprevir, in combination with pegylated interferon alfa and ribavirin (PR). The standard HCV genotype 2/3 antiviral medication regimen remained PR since no DAA were available for these genotypes at the time. To promote generalizability, the specific type or length of antiviral treatment was left to the discretion of the HCV clinical team or to local clinical trials in which participants enrolled. All participants receiving antiviral treatment were monitored for significant adverse events that could lead to early termination of treatment.

### 2.5. Measures

The primary outcomes were treatment initiation and SVR, defined as “undetectable” outcomes of viral testing conducted at 12 weeks (or more) after the end of treatment [28, 29]. A secondary outcome was the proportion of patients completing 100% of planned treatment duration and the proportion of treatment weeks completed (0–100%). All antiviral treatment data was abstracted from participant medical records by a trained research assistant and 100% confirmed by the study data manager. Abstracted data included medication type and dose, treatment duration recommended, and treatment duration attained.

Serious adverse events were also assessed and were defined as any hospitalization, emergency room visit, and/or death. All patients were followed for treatment initiation status through June 2013 at which time the intervention ended. Treatment completion outcomes were followed through December 2013 and the primary outcome of SVR was followed until March 2014.

### 2.6. Statistical Analysis

“Intent-to-treat” analysis was performed for all clinical outcomes. The univariate association between SVR and covariates was examined using simple logistic regression. Despite a limited sample size, multivariable logistic regression was used to study the difference in SVR between intervention and control groups adjusting for baseline covariates. Only variables that were significantly unbalanced between groups and significantly associated with the outcome (*p* < 0.05) were considered as potential covariates for multivariable analysis and were kept in the final model if the *p* value < 0.05. Treatment initiation differences were compared using the log-rank chi-square test and visualized with a Kaplan–Meier curve. Cox proportional hazard model was used for multivariable analysis to adjust for covariates. The actual treatment completion percentage, the proportion of subjects completing 80% of treatment, and adverse events were summarized by treatment group and compared using Wilcoxon rank sum and Fisher's exact test.

## 3. Results 

### 3.1. Study Participants

The flow of patients through the screening, enrollment, and randomization process is shown in Figure 1. Of the 83 participants that provided initial consent, 4 participants refused further participation, were not randomized, and were withdrawn. Thus, 79 participants were randomized, 39 to Usual Care and 40 to Integrated Care. Patients were enrolled over 14 months, and the mean patient follow-up period was 16.6 months (range = 10.3–21.9 months).

Table 1 shows the baseline characteristics for study participants by intervention arm. Participants were 40.5% non-White and had a high frequency of known barriers to access (72% unemployed or disabled, 42% homeless within the prior 5 years, 63% with prior psychiatric illness, and 47% with a prior substance use diagnosis). When considering substance use as a whole, 32% of participants reported active drug use within one year and/or active alcohol abuse based on positive AUDIT-C score. The mean BDI score was 15.5, and 71% met criteria for depression at enrollment. The UC group had a higher mean age (*p* = 0.01) than the IC group. The two intervention groups were not significantly different on any other baseline characteristics.

### 3.2. Treatment Initiation and Time to Treatment Initiation

**Figure 2:**
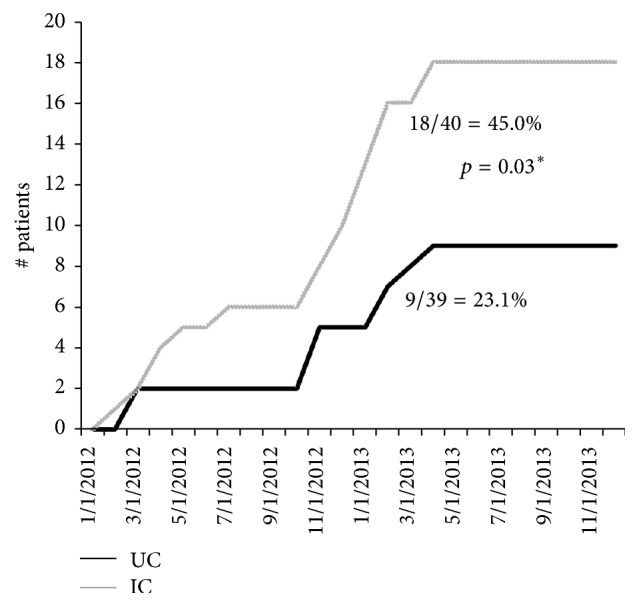
Antiviral treatment initiation over time in Integrated Care (IC) versus Usual Care (UC) groups.

Almost twice as many IC participants (18/40 or 45%) initiated treatment compared with subjects in Usual Care (9/39 or 23%) (*χ*^2^ = 4.59, *p* = 0.032, Figure 2). In multivariate analysis, age was the only variable initially identified for inclusion in multivariable analysis, but, to replicate our prior analysis, we included genotype which was identified as a significant predictor for treatment initiation in our previous study [24]. When age and genotype were added to the model as covariates, time to treatment initiation was not significantly different between IC and UC participants (HR = 1.88, 95% CI = 0.82–4.27, *p* = 0.13). Participants with type 2–4 genotype (HR = 3.24, *p* = 0.01) and younger subjects with (HR = 0.95, *p* = 0.01) started treatment earlier. Of patients that initiated treatment, six patients had genotype 2 or 3 in IC and two patients had genotype 2 in UC; and 1 patient in both IC and UC received experimental DAA therapy.

### 3.3. Sustained Viral Response (SVR)

For patients that initiated antiviral therapy, the rate of SVR was not significantly different (IC: 12/18 = 67%; UC: 5/9 = 55%; *p* = 0.23). When looking only at genotype 1, 7/12 had an SVR in IC, while 4/7 had an SVR in UC.

### 3.4. Intent-to-Treat Sustained Viral Response (SVR)

**Figure 3:**
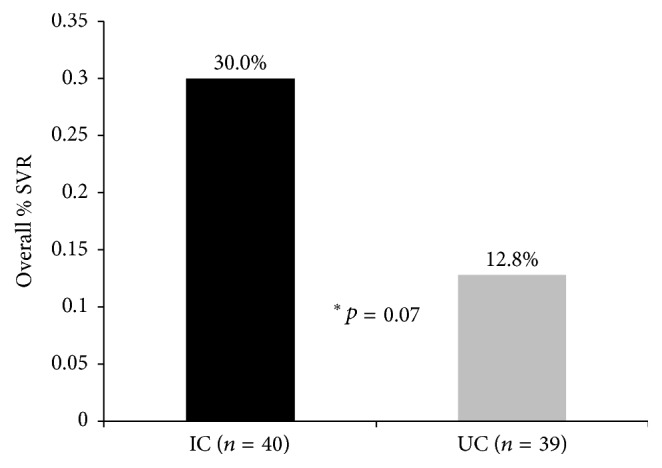
Cumulative probability of treatment initiation by intervention group over time (months). Patients are censored at end of study follow-up. ne = number of events (treatment initiation).

Among all randomized patients, including those that never initiated treatment, the rate of SVR was 2.3 times greater in the IC group (12/40; 30%) than in the UC group (5/39; 13%). The difference trended toward significance but was not statistically significant at *p* < 0.05 (OR = 2.91, *p* = 0.07) in univariate analysis (Figure 3). Age was the only variable identified for inclusion in multivariable analysis but was not significant in the final model (OR = 0.94, *p* = 0.07) (see Table 2).

### 3.5. Treatment Completion

Among patients that initiated antiviral treatment, no significant differences were found between the proportions of IC (14/18 = 78%) and UC participants (5/9 = 56%) completing 100% of planned treatment duration.

### 3.6. Adverse Events

There were no significant differences between IC and UC groups in the number of serious adverse events, measured by hospitalizations, hospital days, emergency room visits, and deaths (Table 3).

## 4. Discussion

Study results indicate that IC participants were twice as likely to initiate antiviral treatment. The proportion of treated participants obtaining an SVR and competing the full duration of planned treatment was not significantly different between groups. When viewing SVR from an intent-to-treat perspective, 30% of all IC participants versus 13% of all UC participants obtained an SVR.

Although new antiviral treatments are demonstrating high SVR rates in clinical trials [1, 2], the treatments are expensive and SVR rates may be lower in clinical practices providing care to patients who face additional challenges [10, 15]. HCV-infected persons who are homeless, lack resources, and/or have ongoing substance use and psychiatric disorders may not readily engage in care or be adequately prepared to adhere to antiviral regimens without interventional assistance. For example, one recent VA study found that almost half of the HCV patients reviewed were poor candidates for interferon-free treatment based on psychiatric and substance use comorbidity [11]. Recently, a study tried to engage 481 consecutive HCV patients at high risk for advanced stage 3-4 fibrosis (FIB4 score > 2.4) in HCV care. Of these, 379 patients were considered medically eligible for antiviral treatment. Within this group, 32% (123/379) were unwilling or unable to engage in HCV-related care after repeated outreach attempts or clinic visits. Factors associated with nonengagement included history of homelessness, substance use disorder, and other comorbidities [12]. In addition, Medicaid payments have been restricted in many states for treating HCV patients with active or recent SUDs with the most efficacious and expensive regimens [14]. Thus, there remains a clear need for programs to better prepare or engage HCV-infected persons with psychiatric and substance use comorbidity for antiviral treatment in the DAA era.

The current study sought to confirm the results of a previous multisite study that demonstrated that HCV Integrated Care could more than double treatment rates and SVR in this population [24]. The previous study was conducted from 2009 to 2012 when the majority of treatments were pegylated IFN plus ribavirin. The current data were collected from 2012 to 2013 as part of a smaller RCT during the era of first-generation DAA triple therapy (telaprevir or boceprevir plus pegylated interferon and ribavirin). The current results were very similar to those of the larger study, with almost twice as many Integrated Care participants initiating treatment with DAA triple therapy regimens, when compared to Usual Care, and 2.3 times as many Integrated Care participants achieving an SVR. Despite the large effects size, the group differences in treatment initiation and SVR did not quite reach statistical significance in multivariate analyses, because of a limited sample size and a smaller than expected increase in treatment rates with first-generation DAAs. However, when viewed as a follow-up to and in combination with the results of our large multisite trial [24], and given the size of effects found in the current study, we conclude that the HCV Integrated Care Program remains an effective tool for boosting treatment rates and SVR in persons with psychiatric and substance use disorders with DAA treatments.

The overall treatment rates with the new first-generation DAA treatments were only slightly higher than those found in the previous study, which is consistent with national VA data showing that treatment rates did not increase significantly with the advent of DAA triple therapies [30]. This likely results from even more challenging side effects when adding a DAA to standard pegylated interferon and ribavirin. For those receiving antiviral therapy, treatment adherence rates and SVR rates tended to be higher for IC participants, but differences were not statistically significant given the relatively small patient numbers. The SVR rates are comparable to those reported from VA national data (50%) for genotype 1 patients treated with first-generation DAA therapies [9]. Thus, although not statistically significant here, these data along with fewer deaths in the UC group follow the same trend found previously, making it unlikely that study effects resulted from healthcare providers simply treating more IC patients. For example, combining data from both studies, we find 9 deaths among 222 IC patients and 17 deaths among 220 UC patients (*p* = 0.04). For veterans with HCV and with a history of substance use and psychiatric disorders, working closely with a mental health case manager may provide more than motivation and support to start antiviral treatment, inclining them to better adhere to treatment and other healthcare recommendations.

A limitation of the current study was the overall sample size. In 2011, a modification to a previous larger multisite study was obtained to conduct the current smaller RCT, capitalizing on existing infrastructure. At that time, there were expectations that treatment rates would increase dramatically given the increased efficacy of the new triple therapies. Thus, the power calculations assumed higher treatment rates. The effect found in the current study would have been statistically significant had the treatment rates been as expected. The lower treatment rates were evident during the current RCT but additional time and resources were not available to extend enrollment. The current sample size also limited efforts to conduct subgroup analyses for patients with substance use disorders versus other comorbidities, or with specific genotypes. An additional limitation is that patients and providers were not blinded to intervention assignment. It is possible that cross-contamination between experimental groups occurred. However, the IC practitioner never interacted with UC comparison group participants, and if physician contact with the IC interventionist influenced care of UC participants, an increase in antiviral treatment would be expected in the UC group, biasing the study toward the null hypothesis and strengthening conclusions.

Overall, the concept of integrated or collaborative care for patients with multiple comorbidities has been shown to be effective in a number of clinical settings [31]. After the initial surge in interferon-free DAA treatment initiation for HCV patients with better access, less comorbidity, or cirrhosis, medical practices may need to focus on patients with additional barriers to care. As demonstrated recently, a significant percentage of patients with HCV most at risk for advanced fibrosis remain reluctant or unable to engage in care with interferon-free treatments [12]. The principles of Integrated Care include colocation of critical resources, multidisciplinary team members, patient activation, and coordination of care targeted to the needs of individual patients. Although the ongoing need for HCV Integrated Care interventions may currently be more limited to subgroups that are difficult to treat, the results presented support the use of Integrated Care strategies for optimizing the numbers of patients that successfully complete antiviral therapy while conserving limited resources. With many HCV-infected persons still needing treatment, efforts to implement Integrated Care can facilitate initiation and completion of interferon-free DAA regimens.

In conclusion, further study of the benefits of HCV Integrated Care interventions is warranted, especially among hard-to-treat subgroups such as IV drug users. The current study confirms the results of a previous multisite study, yet the lack of adequate power was a limitation. Thus, it is important for future studies to ensure the recruitment of a sample size providing adequate power.

## Figures and Tables

**Table 1:** Patient characteristics.

		Total (*n* = 79) *n* (%)	Integrated Care (*n* = 40) *n* (%)	Usual Care (*n* = 39) *n* (%)	*p* value
Demographics					
Age	Mean (SD)	55.7 (8.4)	54.0 (8.7)	57.4 (7.7)	0.01
BMI	Mean (SD)	28.8 (5.5)	29.0 (5.8)	28.6 (5.3)	0.79
Gender	Male	75 (94.9)	37 (92.5)	38 (97.4)	0.62
Race/ethnicity	African American or Black	19 (24.1)	7 (17.5)	12 (30.8)	0.36
	White	47 (59.5)	25 (62.5)	22 (56.4)	
	Native American	1 (1.3)	0 (0)	1 (2.6)	
	Hispanic	11 (13.9)	7 (17.5)	4 (10.3)	
	Asian/Pacific Islander	1 (1.3)	1 (2.5)	0 (0)	
Marital status	Single	20 (25.3)	11 (27.5)	9 (23.1)	0.69
	Married or widowed	25 (31.6)	11 (27.5)	14 (35.9)	
	Separated or divorced	33 (41.8)	18 (45.0)	15 (38.5)	
	Missing	1 (1.3)	0 (0)	1 (2.6)	
Education	Grades 1 to 8	1 (1.3)	0 (0)	1 (2.6)	0.61
	Grades 9 to 11	4 (5.1)	2 (5.0)	2 (5.1)	
	High school/GED	18 (22.8)	12 (30.0)	6 (15.4)	
	Some college	43 (54.4)	21 (52.5)	22 (56.4)	
	College grad	6 (7.6)	2 (5.0)	4 (10.2)	
	Post-grad	1 (1.3)	0 (0)	1 (2.6)	
	Missing	6 (7.6)	3 (7.5)	3 (7.7)	
Employment	Full- and part-time	9 (11.4)	4 (10.0)	5 (12.8)	0.92
	Unemployed	28 (35.4)	13 (32.5)	15 (38.5)	
	Disabled	29 (36.7)	15 (37.5)	14 (35.9)	
	Retired or volunteer	11 (13.9)	7 (17.5)	4 (10.2)	
	Missing	2 (2.5)	1 (2.5)	1 (2.6)	
Homeless in last 5 years	Negative	38 (48.1)	18 (45.0)	20 (51.3)	0.51
	Positive	33 (41.8)	19 (47.5)	14 (35.9)	
	Missing	8 (10.1)	3 (7.5)	5 (12.8)	

Clinical characteristics					
Primary genotype	Type 1	62 (78.5)	29 (72.5)	33 (84.6)	0.27
	Types 2, 3, and 4	17 (21.5)	11 (27.5)	6 (15.4)	
Fibrosis level	Mean (SD)	2.2 (1.8)	2.1 (1.8)	2.3 (1.9)	0.84
Prior liver biopsy		36 (45.6)	18 (45)	18 (46.2)	0.99
Biopsy after enrollment		18 (22.8)	8 (20)	10 (25.6)	0.60
Prior HCV treatment		14 (17.7)	7 (17.5)	7 (17.9)	0.96
Prior psychiatric illness		50 (63.3)	27 (67.5)	23 (59)	0.49
Prior substance abuse		37 (46.8)	19 (47.5)	18 (46.2)	0.99
Other prior comorbidity		34 (43%)	16 (40%)	18 (46.2)	0.65
Number of comorbid disorders	Mean (SD)	0.59 (0.81)	0.6 (0.9)	0.59 (0.72)	0.73

Eligibility screen measures					
Depression (BDI) screen^a^	Negative	22 (27.8)	14 (35.0)	8 (20.5)	0.21
	Positive	56 (70.9)	26 (65.0)	30 (78.9)	
	Missing	1 (1.3)	0 (0)	1 (2.6)	
Depression (BDI score)	Mean (SD)	15.5 (9.2)	15.4 (9.5)	15.7 (9.0)	0.96
PTSD risk screen^b^	Positive	36 (53.7)	21 (56.8)	18 (52.9)	0.81
Alcohol (AUDIT-C) screen^c^	Positive	15 (19.0)	7 (17.5)	8 (20.5)	0.78
AUDIT-C screen score	Mean (SD)	1.5 (2.9)	1.4 (2.5)	1.7 (3.2)	0.83
Active drug use screen^d^	Positive	13 (16.5)	5 (12.5)	8 (20.5)	0.38
Active alcohol risk *or* drug use (past 6 months)	Positive	25 (31.6)	12 (30.0)	13 (33.3)	0.81

^a^BDI screen positive score includes scores ≥ 10; ^b^VA Primary Care PTSD screen score ≥ 3; ^c^AUDIT-C screen positive score ≥ 4 at baseline; ^d^self-reported active drug use and/or positive urine toxicology in 6 months prior to baseline (excluding marijuana).

**Table:** (a) Association between time to treatment initiation and intervention group

Variables	Hazard ratio	95% confidence interval	*p* value
Intervention group, IC^*∗*^ versus UC^†^	1.88	(0.82, 4.27)	0.13
Age	0.95	(0.92, 0.99)	0.01
Genotype (type 2, 3, and 4 versus type 1)	3.24	(1.43, 7.37)	0.01

**Table:** (b) Association between SVR and intervention group

Variables	Odds ratio	95% confidence interval	*p* value
Intervention group, IC^*∗*^ versus UC^†^	2.91	(0.92, 9.27)	0.07

^*∗*^IC = Integrated Care; ^†^UC = Usual Care.

**Table 3:** Adverse event outcomes.

	Integrated Care (*n* = 40) Mean (SD)	Usual Care (*n* = 39) Mean (SD)	*p* value
Number of hospitalization events/patient	0.35 (0.83)	0.31 (0.80)	0.81
Number of hospital days/patient			
Subjects with hospital event^a^	14.3 (23.6)	11.5 (9.01)	0.65
All subjects^b^	2.85 (11.6)	1.77 (5.32)	0.82
Number of ER visits/patient	0.78 (1.12)	0.74 (1.29)	0.71
Number of deaths, *n* (%)	1 (2.5)	3 (7.7)	0.36

^a^14 (8 IC and 6 UC) subjects with hospitalization events; ^b^number of hospital days is 0 for subjects without hospitalization event; IC = Integrated Care; UC = Usual Care.
